# Comparative Analysis of the Silver Nanoparticle’s Yield for Pico-Femto-Nanosecond Laser Generation

**DOI:** 10.3390/mi14061220

**Published:** 2023-06-09

**Authors:** Alena Nastulyavichus, Sergey Kudryashov, Andrey Ionin

**Affiliations:** 1 P. N. Lebedev Physics Institute, Russian Academy of Sciences, 119333 Moscow, Russia; 2 School of Natural Sciences and Mathematics, Ural Federal University, 620000 Ekaterinburg, Russia

**Keywords:** silver nanoparticles, laser ablation in liquid, plasmonic resonance, interband transitions, nanoparticles productivity, universal quantitative criteria

## Abstract

Comparative analysis of different laser regimes of silver nanoparticle generation in water was performed for laser pulsewidth in the range of 300 fs–100 ns. Optical spectroscopy, scanning electron microscopy, energy-dispersive X-ray spectroscopy and method of dynamic light scattering were used for nanoparticle characterization. Different laser regimes of generation were used with varying pulse duration, pulse energy and scanning velocity. The proposed universal quantitative criteria as productivity and ergonomicity of the obtained colloidal solutions of nanoparticles were investigated to compare different laser regimes of production. The efficiency per unit energy for picosecond generation of nanoparticles, free from the influence of nonlinear effects, turns out to be higher by 1–2 orders of magnitude than for nanosecond generation.

## 1. Introduction

Nanoparticles (NPs) have great popularity in most fields due to their unique physical and chemical properties which are due to their high surface area and nanoscale size. The properties of the nanoparticles are quite different from the bulk materials due to the small size and high surface area to volume ratio of the nanoparticles. The main idea is not only on the production of nanoparticles but also on the control and manipulation of their sizes [1]. Nanoparticles are suitable candidates for various commercial and domestic applications, which include catalysis, imaging, medical applications, energy-based research, and environmental applications [2].

The generation of a high amount of colloidal nanoparticles of different materials is well established by conventional methods such as wet chemical synthesis or mechanical milling and grinding [3,4]. Since the 1990s, laser ablation has become a promising method for the synthesis of nanoparticles and an industry that has attracted attention [5,6,7]. Pulsed laser ablation in liquids (PLAL) is an attractive method to synthesize nanoparticles due to the simplicity of the procedure and high chemical purity of the produced nanoparticles [8]. This method has some advantages, such as the material variety (metals, semiconductors, polymers, etc.), the high purity of the produced nanomaterial, and the dispersion of the nanoparticles in a variety of liquids allowing safe and stable handling of the colloids [9]. The complexity and multiscale nature of PLAL makes it difficult to untangle the various processes involved in the generation of nanoparticles and establish the dependence of nanoparticle yield and size distribution on the irradiation parameters [10].

This method allows you to achieve a productivity of several grams per hour. At present, there is a struggle for the performance of nanoparticles. This is facilitated by the improvement of installations [11,12], the variation of the target geometry [13], thanks to which they were able to achieve a productivity of ~mg/h. There is also information about the productivity of the order of 1 g/h, however, these studies are based on the extrapolation of the ablation rate in seconds or several minutes [3,14]. The work [15] says that metals can be ablated for more than 1 h and that the productivity is 5–20 mg/h, depending on the material. These studies showed that the ablated mass is not proportional to the ablation time, indicating that extrapolation from seconds and minutes does not provide reliable information. The speed of galvanoscanners is limited (about 10 m/s). However, polygon scanners allow scanning at a speed of more than 50 m/s [16]. In addition, their use allows us to overcome the limitations associated with cavitation bubbles and increase the repetition frequency from 50 kHz to 10 MHz. The productivity for such a system was more than 4 g/h [12]. According to the work [17], femtosecond laser ablation in water is 20% more efficient than picosecond laser ablation, but due to higher picosecond laser power, the nanoparticle productivity at the same pulse fluence is three times higher for picosecond laser ablation. According to the literature data, ablating silver in the water by ultrashort laser pulses (fs duration) gains nanoparticle productivity of 7.9 mg/h. In the picosecond time region, maximum productivity increases because of higher available laser power and reaches 8.6 µg/s (31 mg/h) for silver in water surrounding despite that ablation efficiency is higher for femtosecond pulses (2 µg/J for femtosecond irradiation compared to 1.5 µg/J for picosecond ablation). In addition, at the same pulse fluence, the production rate of nanoparticles with picosecond laser of 7.4 g/s is 3.4 times higher compared to femtosecond laser ablation of 2.2 g/s. Authors believe that this is mainly due to the higher power higher repetition rate applied to the sample. In addition, it can be explained by the fact that picosecond pulses compared to femtosecond pulses do not induce strong nonlinear effects leading to pulse disturbances [17]. For laser ablation in the air, there is a comparison of different laser modes [18]. According to this paper, the fs laser has the best performance (efficiency). They used three lasers (4 ns, 25 ps, 150 fs). Productivity was calculated by the volume of the crater. In [19,20], the effect of focusing conditions and pulse energy on the ablation efficiency is studied. According to [21], the highest efficiency in laser ablation of copper and silver targets in a liquid for a wavelength of 1064 nm is observed at higher fluences, and for a shorter wavelength (532 nm) at lower fluences. The process of self-absorption of laser radiation by colloidal nanoparticles also has a great influence on efficiency. There are two possible processes for self-absorption. The first one is “interpulse” self-absorption, in which particles formed by earlier pulses remain in the path of laser radiation and absorb later pulses. The other is “intra-pulse” self-absorption, in which particles produced in an earlier part of one pulse immediately absorb photons from a later part of the same pulse. The latter process must be taken into account when nanosecond laser pulses are used to ablate metals since the release of the ablated substance starts on a picosecond scale [18]. Many works investigate the effect of wavelength on performance [21,22].

It is known that for nanosecond laser ablation, the performance drops from 19 to 150 ns [23]. When laser radiation interacts with a metal target, the laser energy first heats the electrons. The excited electrons transfer their energy to the lattice due to collision processes. Due to the large difference in mass between the electrons and the lattice, the energy transfer requires several picoseconds. Therefore, for pulse durations below the electron–phonon coupling time for a certain material, physical processes occur more or less sequentially, while at ns durations they overlap [24]. As the plume forms, the energy is dissipated into the surrounding liquid, which leads to the formation of steam at the boundary with the water layer. These processes have been widely studied theoretically [10,25] and experimentally [26]. During fs, ps ablation, laser energy is converted into plasma. Compared to subpicoseconds and ns where laser energy partially supports the plasma that occurs at high plasma lifetimes. However, when the critical electron density in the plasma is reached, the laser beam can no longer penetrate the plasma and is reflected, leading to a decrease in the ablation efficiency [23]. In this study, comparative studies of short- and ultrashort-pulse LAL of bulk samples of silver in the water were performed in terms of ablation rate and NPs yield versus laser energy, pulsewidth, and exposure per spot.

These studies were focused to identify optimal laser parameters for maximal total NP yield and its power-specific throughput, using the product of spectrally dependent extinction coefficient of colloidal solutions and their volume as a non-universal, but convenient productivity parameter, characterizing both ablated mass and NP yield.

## 2. Materials and Methods

**Figure 1:**
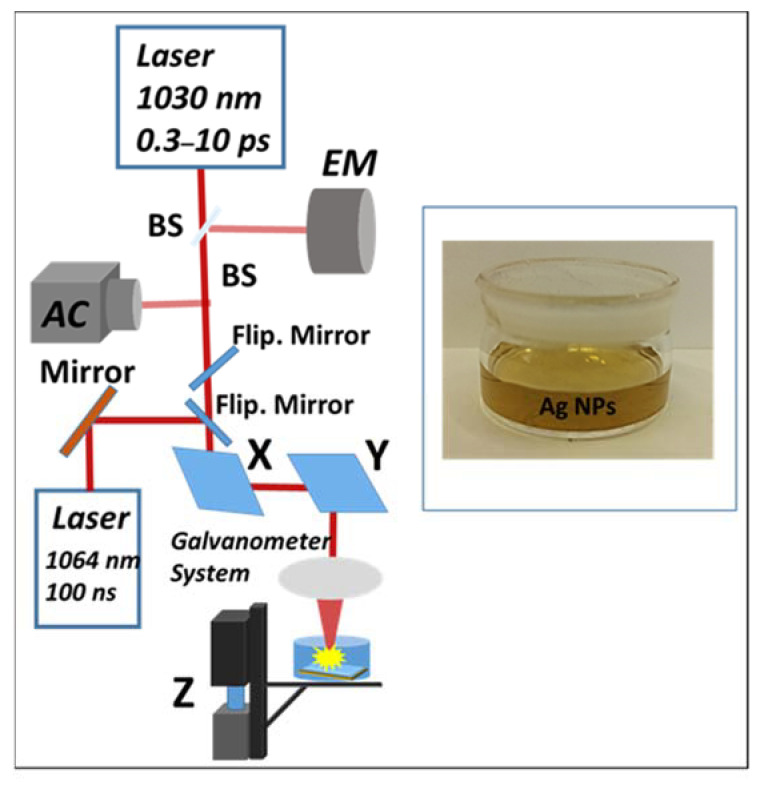
Experimental scheme.

In these studies, nanoparticles generation by method of laser ablation in liquid are used: ytterbium-doped fiber laser Satsuma (central wavelength λ = 1030 nm, maximum output energy E_max_ up to 10 μJ in the TEM00 mode, FWHM pulsewidth τ = 0.3–10 ps, repetition rate f = 0–500 kHz), and nanosecond fiber laser marker HTF MARK (Bulat) on Yb3 + ions (λ = 1064 nm, FWHM pulsewidth of 100 ns, maximum pulse energy Emax = 1 mJ, and pulse repetition rate f ≤80 kHz (Figure 1). The laser beam was focused by a galvanoscanner with an objective focal length ≈100 onto Ag target placed on the bottom of a cuvette and immersed in 3 mL of deionized water (height above the target ≈ 1 mm). Several ablation regimes with different effective numbers of pulses at the point N = 0.5–20 pulses and pulse energy (E = 3.5–5.5 µJ for fs-ps laser generation and 0.3–0.6 mJ for ns) were investigated. Pulse repetition rate was fixed and equal to 20 kHz. The minimum size of the focusing spot (1/e-diameter) D = 20 μm for fs/ps-laser pulses and ≈30 μm for ns- laser pulses. The size of the scanning area was 10 mm × 20 mm.

Transmission spectra of the colloidal solutions were recorded by means of a UV-IR spectrometer (SF-2000, OKB Spectr, Saint-Petersburg, Russia) in the range 190–1100 nm. Scanning electron microscopy (SEM) visualization of the produced nanoparticles was performed using a Tescan Vega microscope, equipped with a STEM detector and energy-dispersion X-ray spectroscopy (EDX) module Aztec One (Oxford Instruments, Abingdon, UK) for chemical micro-analysis of Ag nanoparticles at 10-keV kinetic electron energy. Nanoparticle size distributions were analyzed by means of a dynamic light scattering analyzer Photocor Compact. Mass loss was investigated using the microbalance AND BM-20 (AND, Tokyo, Japan).

## 3. Results and Discussions

### 3.1. Evaluation of the Ag NPs Generation Efficiency

The optical transmission of the obtained colloidal solutions was studied in the range of 200–1000 nm. Then the transmission was converted to the extinction coefficient. It is known that colloidal silver nanoparticles have two absorption peaks: a weak absorption peak is located at the UV light range of ≈200–300 nm, due to interband transition, and a strong absorption peak is located at ≈400 nm wavelength due to surface plasmon resonance [22,27].

**Figure 2:**
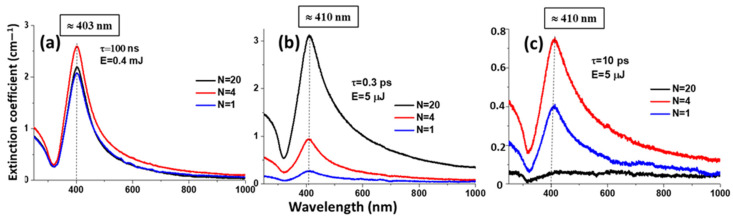
Extinction coefficient of silver colloidal solution obtained (**a**) for pulse duration 100 ns, (**b**) 0.3 ps, (**c**) 10 ps for different laser exposures.

The main characteristic feature of the curves is the localized plasmon resonance of the silver nanoparticles at the characteristic wavelength of ~410 nm. As an example, Figure 2 demonstrates the extinction coefficient for colloidal silver solutions obtained by the method of laser ablation in the water of the Ag bulk target for different numbers of pulses per point (exposures).

In this work, to study the efficiency (productivity) of the generation of silver nanoparticles, we used a criterion based on measurements of the extinction coefficient in the region of interband transitions and localized plasmon resonance. The extinction coefficient in this region provides information on the mass of nanoparticles, which was confirmed in our work by an additional method—measurement of the mass yield. This criterion makes it possible to compare the efficiency of nanoparticle generation for different laser systems. The extinction coefficient in the region of interband transitions does not depend on the shape of nanoparticles and, therefore, can be compared with the amount of removed mass. Since different works use different pulse repetition rates and scanning speeds, for convenient comparison, it is necessary to consider the results in terms of one pulse. To confirm this criterion, the mass loss per impulse is used. Another comparison criterion that was considered in this paper is energy efficiency, i.e., efficiency per unit of energy spent on the generation of a colloidal solution and the corresponding mass loss.

**Figure 3:**
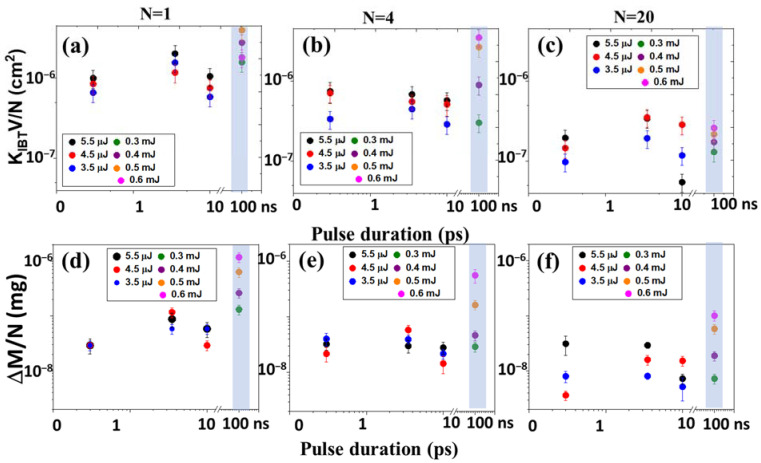
Dependence of the extinction coefficient (K_IBT_ × V)/(N) (**a**–**c**) and mass loss M/N (**d**–**f**) for differ number of pulses (N) on the pulse duration of laser radiation.

**Figure 4:**
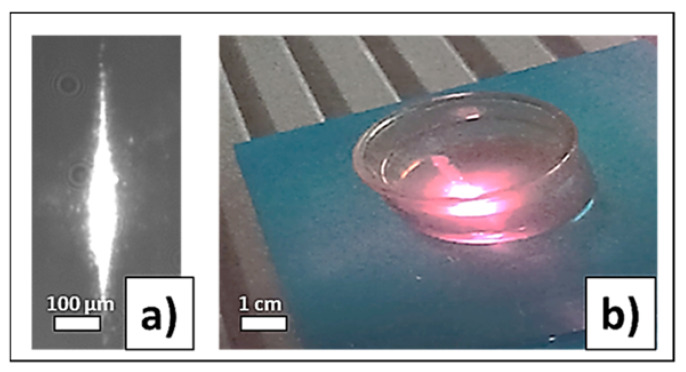
Visualization of plasma channel (**a**); plasma (**b**) in distilled water.

Figure 3a–c demonstrates the dependence of the of nanoparticle generation efficiency (the product of the extinction coefficient in the region of interband transitions (300 nm) and the solution volume per pulse for different exposures (N:1, 4, 20) on the pulse duration of laser radiation). With an increase in the pulse duration of laser radiation in the range of subpico-, picoseconds, the efficiency initially increases, then a local maximum is observed in the region of several picoseconds, after which the efficiency decreases. The decrease in efficiency in the region of subpicosecond durations is associated with the appearance of nonlinear effects—self-focusing and filamentation (the visualization of the plasma channel observed for a pulse duration of 0.3 ps in distilled water is shown in Figure 4a) [28]. Similar behavior was observed earlier for the efficiency curves for the generation of gold nanoparticles [29].

The decrease in efficiency in the region of several picoseconds is associated with the acoustic unloading of the heated layer during heating by the pulse due to rarefaction waves, in which the thermal expansion of the heated substance occurs [30,31,32]. The ablation regime for nanosecond generation is associated with the phase explosion mechanism (homogeneous boiling of an overheated liquid in the near-critical region) and subcritical erosive plasma; for subpico-picosecond ablation, the main contribution is made by the phase explosion mechanism (expansion of supercritical fluid).

In this case, the efficiency for nanosecond laser pulses is almost an order of magnitude higher. A similar trend is also observed for the target mass loss (Figure 3d–f), which shows that an almost constant proportion of the ablated substance passes into the colloidal solution.

**Figure 5:**
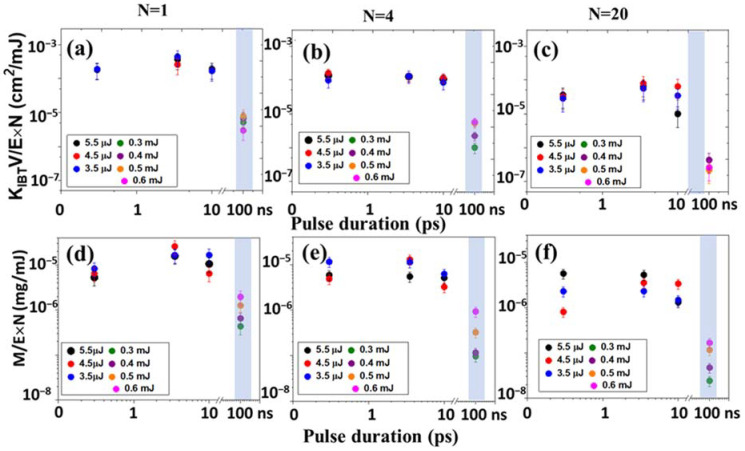
The energy efficiency dependence (K_IBT_ × V)/(E × N) (**a**–**c**) and mass loss per unit of energy M/(E × N) (**d**–**f**) on the pulse duration of laser radiation.

To estimate the energy efficiency of the process (ergonomicity) of generating silver nanoparticles, we considered the efficiency (K_IBT_ V/N and the loss of mass M per unit energy (E) spent on the generation of a colloidal solution (K_IBT_ V/E × N and M/(E × N) (Figure 5).

In terms of energy efficiency of the process of generating nanoparticles, picosecond laser pulses, which are free from the influence of nonlinear effects, provide efficiency in terms of the unit energy of the radiation pulse by 1–2 orders of magnitude higher than for the nanosecond pulse duration. A strong effect in the region of nanosecond pulse durations is associated with the appearance of subcritical erosive plasma, which shields the surface (Figure 4b).

Then, the dependences of laser generation efficiency on the pulse energy were investigated for the three pulse durations. The extinction predominantly increases with increasing energy and decreases with the increasing number of pulses per point (decreasing the scanning speed). The mass loss for the pulse durations of 0.3 and 10 ps behaves differently: at first, it falls, then begins to grow. As for the nanosecond mode, in this case, the dependencies behave in a similar way with the extinction, except for the single-shot pulse mode.

**Figure 6:**
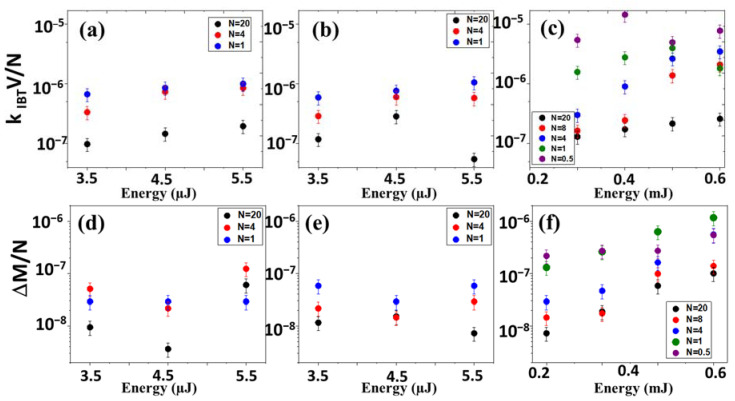
Dependence of laser generation of Ag NPs efficiency and mass loss on the pulse energy for differ number of pulses (N) (**a**,**d**) 0.3 ps, (**b**,**e**) 10 ps, (**c**,**f**) 100 ns.

With an increase in energy, a monotonous increase in efficiency and mass loss is also observed in the range of pulse durations: 0.3–10 ps (Figure 6), which corresponds to the literature data [3].

With an increase in the number of pulses per point, the efficiency of nanoparticle generation and mass loss decrease, this is due to the fact that for low scanning speeds in the region of large overlap on the target surface of the next laser pulses, the interaction of the laser beam with previously ablated nanoparticles and the previously generated cavitation bubble is the main mechanism, hindering productivity gains. The cavitation bubble contains an extremely high concentration of primary nanoparticles, which can scatter, reflect, or absorb subsequent laser pulses, which drastically reduces the efficiency of ablation.

### 3.2. Size Characterization and SEM Visualization of Ag NPs

**Figure 7:**
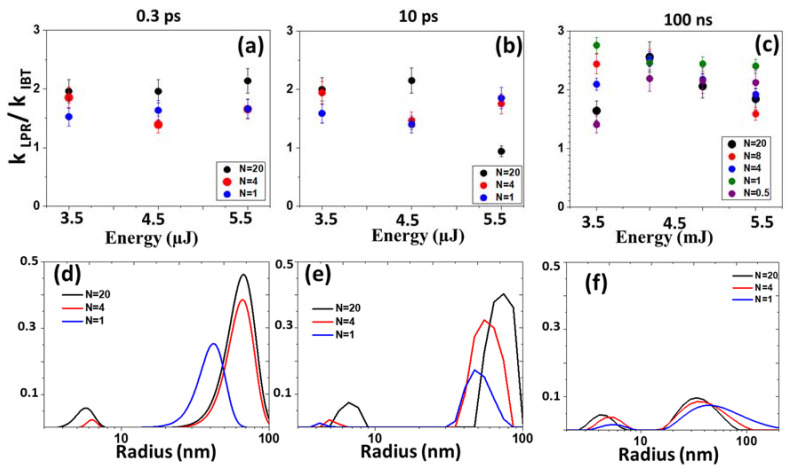
Normalized extinction coefficient K_LPR_/K_IBT_ of Ag NPs colloidal solutions versus laser energy at the different pulse widths: 0.3 ps (**a**), 10 ps (**b**), and 100 ns (**c**) and corresponding particle size distributions (**d**–**f**).

**Figure 8:**
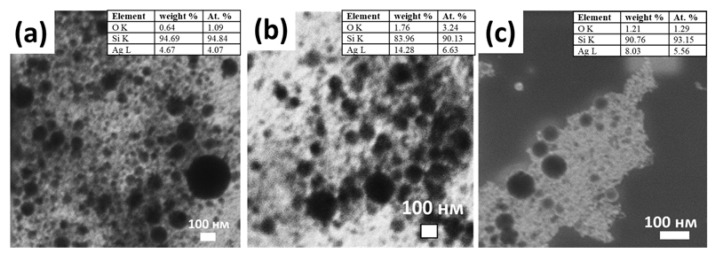
SEM visualization of Ag NPs for pulse duration 0.3 ps (**a**), 10 ps (**b**), and 100 ns (**c**) insets: EDX results.

The dynamic light scattering technique used for determining the NPs sizes demonstrates the presence of nanoparticles with sizes from 5 to 150 nm (Figure 7d–f). Nanosecond generation is characterized by a wider distribution. These data were confirmed by SEM-visualization (Figure 8). As the laser scanning speed increases, the sizes increase for all laser pulse durations. Normalized extinction coefficient—the ratio of the extinction coefficient in the region of localized plasmon resonance (≈405 nm) to the extinction coefficient in the region of interband transitions (300 nm) (K_LPR_/K_IBT_)—provides information on the dispersion of nanoparticles (Figure 7a–c).

For the obtained nanoparticles, this indicator varies from ≈0.9 to 3. For a pulse duration of 0.3 ps and 10 ps, it practically does not change, which indicates the constancy of the particle sizes. The smallest nanoparticles were synthesized by the nanosecond laser [27]. For nanoparticles obtained as a result of laser ablation in the range of durations of subpico-, pico-, and nanoseconds, a spherical shape is characteristic (Figure 8).

According to the EDX data, the nanoparticles obtained for different pulse durations are characterized by slight oxidation (0.64–1.76%) (silicon—the substrate on which the nanoparticles were deposited).

## 4. Conclusions

The efficiency of colloidal silver nanoparticles generation for IR laser pulses in the range of subpico-, pico-, and nanosecond pulse durations was studied. To analyze the efficiency of laser generation of silver nanoparticles at comparable parameters of laser systems with different pulse durations, a comparison quantitative criteria criterion was proposed (the product of colloidal solution volume and the extinction coefficient in the region of interband transitions) in terms of a pulse and a unit of radiation energy. Mass loss per pulse was used to validate the results. It was shown that the highest efficiency of laser generation of silver nanoparticles for a wavelength in the near IR range (wavelength ≈ 1 mm) and a pulse repetition rate of 20 kHz with comparable scanning parameters in a liquid is observed for nanosecond ablation limited by the formation of subcritical erosive plasma. At the same time, the efficiency per unit energy for picosecond generation, free from the influence of nonlinear effects, turns out to be higher by 1–2 orders of magnitude than for nanosecond.

## Data Availability

The related data are available from the corresponding author upon a request.
